# Communication models in a foreign language in relation to cognitive style category width and power distance

**DOI:** 10.3389/fpsyg.2023.1272370

**Published:** 2024-01-08

**Authors:** Dasa Munkova, Eva Stranovska, Michal Munk

**Affiliations:** ^1^NLP Lab, Department of Informatics, Constantine the Philosopher University in Nitra, Nitra, Slovakia; ^2^Department of Romance and German Studies, Constantine the Philosopher University in Nitra, Nitra, Slovakia; ^3^Science and Research Centre, University of Pardubice, Pardubice, Czechia

**Keywords:** foreign language, communicative competence, cognitive style, power distance, request modeling

## Abstract

**Introduction:**

Understanding how category width of cognitive style and power distance impact language use in cultures is crucial for improving cross-cultural communication. We attempt to reveal how English foreign language students, affected by high-context culture, communicate in English as a foreign language. What models of foreign communicative competence do they create?

**Methods:**

We applied association rule analysis to find out how the category width of cognitive style affects the foreign communication competence in relation to culture and language.

**Results:**

The requester tends to be more formal and transfers conventional norms of the culture of the mother tongue into English, which mainly affects the use of alerters and external modifications of the head act of request.

**Discussion:**

A broad categorizer, regardless of social distance, prefers to formulate the request in a conditional over the present tense form, contrary to narrow categorizers who, in a situation of social proximity, prefer the request form in the present tense. A similar finding was shown in the case of external modifications of the head act, where we observed the inversion between broad and narrow categorizers, mainly in the use of minimizers and mitigating devices.

## 1 Introduction

Language learning, such as other behavioral learning, seeks to explain how human beings respond to certain stimuli. Language learning is more than a cognitive ability, similar to the ability to understand symbols, recognize patterns, and deduce from previous experiences (e.g., Brown, 2018 or Goldberg, 2019). Language as a tool for communication was privileged, while the meaning of oral utterance (speech) in actual use along with its function was prioritized over the acceptability of the text—perfectly written sentences (Kanwit and Solon, 2022). The level of language comprehension and speech production is related to the knowledge of language rules (Hymes, 1967, 1972). Communicative competence describes the knowledge that the speaker (e.g., requester) and listener (e.g., requestee) have to communicate appropriately in different social situations. It reflects not only the knowledge of the rules of understanding and production of language but also its social meaning (Hymes, 1992). Speakers’ knowledge of linguistic and sociolinguistic rules, as well as their ability to use this knowledge of language rules in interaction (Whyte, 2019), distinguishes the communicative competence from linguistic performance. Communicative competence covers grammatical competence (knowledge of the grammatical rules and lexicon), sociolinguistic competence (rules of language use and rules of discourse), and strategic competence ((non-)verbal communication strategies) (Canale and Swain, 1980). Over time, other definitions emerged, which, however, are only a modification of the initial theory of communicative competence (e.g., Celce-Murcia et al., 1995; Pulido and Pérez, 2004; Savignon, 2017; Schauer, 2021; Kanwit, 2022). Based on these theories, many models of communication competence were created. For example, a model of communication competence (Celce-Murcia et al., 1995) comprises five core competences: linguistic, strategic, sociocultural, discourse, and actional competence. The last-mentioned core competence consists in the transmission and understanding of the communication intention, i.e., it represents an extent of match between actional intent (speech acts) and linguistic form (speech act elements). Communicative competence covers three types of activity: communication as influencing the opinion or position of the interlocutor; cooperation; awareness of the spoken and perceived content of the thought (message) (Spencer-Oatey and Franklin, 2009).

In the context of language learning, the emphasis is placed on the conveyance of meaning over the appropriateness of speech in learning a (foreign) language (Canale and Swain, 1980). The learner must first be given the opportunity to engage in communication in real situations and only then focus on the structure of speech and the selection of appropriate language means. Lexical and grammatical knowledge is not the basis for achieving communicative competence and its individual sub-competences (Glaser and Limberg, 2020). Language is best taught when it is being used to transmit messages and not when it is explicitly taught for conscious learning (Krashen and Terrell, 1983).

### 1.1 Foreign language communicative competence

Communicative competence in a foreign language is a set of skills, knowledge, attitudes, and communicative experience that is necessary for understanding others and achieving communication goals (Balanaieva et al., 2023) but also as an integral personal and professional quality of a person with a certain level of language (Vasilieva, 2020). Communicative competence in a foreign language can be considered as (1) a target of second language acquisition, (2) a main goal of second language teaching and learning, or (3) the object language testers seek to measure via performance tests (Whyte, 2019).

In the context of foreign language learning, culture plays a vital role in foreign language communicative competence (Turko et al., 2021). Students are progressively exposed to the target language and culture, gradually forming a cognitive anchor for language and cultural understanding. Students tend to use their language in accordance with their cognitive style (Kashima et al., 2014). Culture can be conceptualized from three equal perspectives: (a) ways of doing things, (b) ways of thinking and feeling, and (c) ways of talking (communication) (Sinha, 2021). Through a language, the culture is transmitted and maintained, and vice versa, culture helps us understand the given language better (Kreiner, 2019). Foreign language learning is a transformative process, involving a transition from one culture to another, fostering an understanding of diverse cultural patterns, encompassing both the cultural background of one’s mother tongue and the awareness of communication styles within the target culture (Výrost and Slaměník, 2008).

The most prominent distinction in communication lies in the contrast between direct and indirect communication approaches (De Mooij, 2014). De Mooij (2014) highlights the importance of culture in shaping communication models and styles and how understanding this is essential when considering communication within different cultural contexts. The relationship between language and culture is shown not only by external elements and processes but also by internal elements and processes, i.e., cognitive representation of language (Altarriba and Basnight-Brown, 2022). Hofstede (2001) identified four dimensions of natural culture: power distance (PDI), uncertainty avoidance (UAI), individualism vs. collectivism (IDV), and masculinity vs. femininity (MAS). Hofstede’s work on culture provides insights into the dynamics of cross-cultural relationships. He showed that Slovakia belongs to an extreme outlier in two of the dimensions: power distance (PDI) and masculinity (MAS). The PDI dimension expresses the degree to which the less powerful members of a society accept the fact that power is distributed unequally (Hofstede, 2001). Hierarchy index/power distance/power proximity measures the extent to which the members of organizations and institutions expect, accept, and approve higher or smaller inequality of power positions in society; it shows how such culture appreciates, acknowledges hierarchy, and shows respect to authorities. A high index of power indicates natural acceptance and expectations that the power distribution is unequal. Normally, such cultures demonstrate wide social differences and high levels of importance are placed on social status. Low-index cultures emphasize equality of chances for every individual. They display low hierarchy structures. Within the MAS dimension, the pole of masculinity reflects a society with a stronger preference for achievement, heroism, assertiveness, and material rewards for success (Bašnáková et al., 2016). Slovaks are more power distant and institutionally collectivistic (Pucko et al., 2013). Slovaks are non-confrontational in communication, listen well, interrupt foreigners only occasionally, and are polite, and they are more punctual than other Slavs (Lewis, 2006). Slovaks lean toward dialogue-oriented cultures, similar to Italian or Arab cultures (Čiefová, 2017). A high level of PDI can be explained as the state when an unequal distribution of power is largely accepted and expected in a society (Hofstede and Hofstede, 2017). Due to a high PDI score, people in Slovakia understand and accept hierarchy in society. On the contrary, people in English-speaking countries with a lower PDI (compared to Slovak) believe that inequalities among people should be minimized; they do not highlight personal achievements, and they do not pay much attention to academic titles. Instead, they address people by their first names and make the atmosphere more personal and informal (Welnitzová and Jakubičková, 2020). Communication style is always influenced by the PDI of the relevant culture (Hofstede et al., 2010). Based on the importance of context in communication, the Slovak culture belongs to a high-context culture, i.e., communication focuses on underlying context, meaning, and tone in the message, and not just the words themselves (Hall, 1976), while similarity is an important characteristic of the culture (requestee and a requester think in the same way). On the other hand, the UK culture is characterized as a low-context culture, i.e., communication is explicitly stated (on explicit verbal skill) to properly understand a message (Hall, 1976) and is characterized by diversity (focus on requester’s needs). High-context cultures prefer oral communication, while low-context cultures favor written communication.

Foreign communicative competence is a topic usually covered by research focusing either on the interpretation of the concept of foreign language communicative competence or on issues related to the development of students’ foreign language communicative competence in the context of second language teaching and learning, or on activities developing students’ foreign language communicative competence in the context of second language acquisition (e.g., Long, 2007; Skehan, 2009; Spada et al., 2015). The last-mentioned research area is the most frequently researched and discussed, especially in the context of ICT use and virtual reality (e.g., Arnó-Macià, 2014; Jung, 2014; Cai Y. et al., 2021; Cai J.-Y. et al., 2021).

Current research on communication models in foreign language learning focuses on the investigation of pragmatic competence within the communicative competence and their impact on the appropriateness of the choice of linguistic and non-linguistic indicators in communication (e.g., Huang, 2019; Glaser and Limberg, 2020; Yan, 2022), on the influence of the mother tongue communication patterns on the communication style in a foreign language (e.g., Fernández and Cairns, 2017), on the influence of the sociocultural environment on pragmalinguistic competence (e.g., Mao, 2021; Mao and He, 2021), on transcultural communication through the media and cultural proximity (e.g., Schulz et al., 2023), on language modeling through ICT (e.g., Verbeke et al., 2017), and on the dynamics of creating models of students’ thinking in a foreign language with regard to the social environment, social motivation, and experience with the culture of the foreign language (e.g., Gehlbach et al., 2016; Kroll and Dussias, 2017; Mao and He, 2021).

We attempt to reveal how a student, affected by high-context culture, communicates in English as a foreign language, and/or what models of foreign communicative competence the student creates. Is their communication intention in the speech act of request influenced by their category width of cognitive style?

In our study, it is a speaker (a student) who communicates in a foreign language (in English) with a person who is socially distant from her/him (a university professor). By formulating a request in a foreign language, we examine the speaker’ knowledge of foreign language rules, her/his ability to use rules to interact, and actual language use during requests (social proximity and distance) involving a person with social power. In addition, we attempt to answer whether the communicative competence in a foreign language is influenced by the width of categorization of cognitive style.

### 1.2 Cognitive style category width

Cognitive style refers to the way an individual thinks and processes information. It is defined as a stable and permanent characteristic of a person, which has an impact on a person’s attitudes, values, and social interaction. Cognitive styles influence appropriateness of behavior toward achieving a goal (Sarmany-Schuller and Šimúth, 2006), in our case to fulfill a request. Categorization has proved to be a suitable variable to measure performance and skill because it allows us to treat different things as if they were identical (Massaro and Ferguson, 1993). Category width is a range of instances included in a cognitive category (Pettigrew, 1982). It reveals individual differences in categorization strategy with two extremes—the broad categorizer who can better apply holistic strategies as opposed to the narrow categorizer who is better in detail analytical information processing (Massaro and Ferguson, 1993).

The process of speech production relies on the utilization of communication rules, involving rule selection, acquisition, and application. The choice of these rules in speech production is influenced, in part, by the width of information categorization within an individual’s cognitive structure. Some individuals only perceive one fixation, for example, lexical, focus on the meaning only; other fixations, such as morphological or syntactical accuracy or phonological awareness, remain filtered. In the context of second language acquisition, the broad categorizers make more errors of overgeneralization and the narrow categorizers formulate more rules than necessary (Salvisberg, 2005).

Investigation of cognitive styles appears in studies on personality as well as cognitive processes (e.g., Sarmány-Schuller, 2011; Grežo and Sarmány-Schuller, 2015; Prokopčáková, 2015; Liu et al., 2016), together with the investigation on the communication of an individual in a foreign language (e.g., Stranovská et al., 2012).

To our knowledge, no studies have dealt with models of foreign communicative competence based on cognitive style category width. Mostly cognitive style is connected to the process of second language teaching and learning (Cai Y. et al., 2021; Cai J.-Y. et al., 2021) or is related to foreign language proficiency (Supriyadi et al., 2020) or to English as a Foreign Language (EFL) learners’ performance in the speech act (Maibodi and Dehghani, 2020); therefore, this study attempts to fill this gap in the literature and research. The contribution of our study lies in the identification of the behavior of an individual (from a high-context culture), using cognitive style category width and communicative competence (language, social, and expressive factor) in speech act of request in a foreign language characterized by a low-context culture. Whether there is an impact of cognitive style category width on the speech act of request among the Slovak learners of English as a foreign language.

### 1.3 Research questions and hypotheses

Understanding how category width of cognitive style and social power and distance impact language use in foreign cultures is crucial for improving cross-cultural communication and fostering cultural sensitivity. This study aims to investigate how an individual’s category width of cognitive style and social power and distance within a foreign culture influence their communication patterns in a foreign language. We attempt to find answers to the following research questions:

To what extent is the foreign communication competence affected by the width of categorization (narrow vs. broad) in target low-context culture? To what extent does the foreign communication competence reflect the students’ width of categorization (narrow vs. broad) in English language?

We state the null hypothesis:

H0: *Cognitive style category width affects foreign communication competence in terms of the use of social, language, and expressive factors when modelling requests*.

In addition to the width of categorization of cognitive style itself, we are interested in whether social proximity and distance also have an impact on communicative competence in a foreign culture. We ask the question to what extent the communicative competence is affected by power distance (D- or D+) in a foreign culture. To what extent does a student’s communicative competence reflect power distance (D- or D+) in a foreign language?

We state the null hypothesis:

H0: *Social Distance affects the language in terms of the use of social, language, and expressive factors when modelling requests*.

The rest of the study is structured as follows. In the next section, we briefly describe social power and distance and their relations to culture and language. Subsequently, we present the methodology of the experiment, including the description of participants and methods applied, which then follows Section 4. Finally, we discuss our findings and summarize our contribution in Section 6.

## 2 Social power and distance

Communication competence deals with three aspects—knowledge, the ability to use, and the ability to adapt to various contexts. The last aspect is related to social variables, such as social distance and power, which affect communication competence not only in the mother tongue but also in a foreign language. Social variables may affect not only the choice of politeness strategies but also the sequential structure of the discourse (Blum-Kulka, 1997). Power is the capacity of an individual to influence the behaviors, thoughts, and/or feelings of others. Power operates on individual, cultural, and structural levels, as well as in our interpersonal relationships (Gerber and Murphy, 2023). In a high-power distance culture (hierarchy culture), such as Slovak, inequality is accepted, contrary to a low-power distance culture, such as English, in which inequality is thought to be unsatisfactory (Hofstede, 2010). The essential element in communication is not the social power but social distance (Díaz-Pérez, 2003). Social power is considered to be a clearly defined hierarchical relationship; the communicating partners apply acquired knowledge, whereas social distance is binary (known vs. unknown) and is defined differently in every culture. Social distance is not a hierarchical type of relationship as opposed to social power. Social distance relates to the image of proximity (close/familiar and distant/stranger), differing from culture to culture, applied in language (Díaz-Pérez, 2003; Spencer-Oatey, 2012). Cultural norms are the most important factors of social distance (Hall, 1976; Sorokowska et al., 2017). Social distance applies to the distance perceived by an individual between herself/himself and her/his listener in a specific situation, working effectively through a common sociolinguistic medium (Díaz-Pérez, 2003). In situations of social distance, people tend to speak more slowly when they address strangers than when addressing friends (Yuan et al., 2006). Language is more involved when social distance is small and more complex and explicit when social distance is larger (Koppen et al., 2019). It is, therefore, more significant in speech production than in social power. Manifestations of social distance have a great impact on the image of communication partners (Fráterová, 2011 or Trubačová, 2016).

The later models of communicative competence (Saleem et al., 2021) have integrated pragmatic competence with two types of knowledge—pragmalinguistics and sociopragmatics—because of its vital role in providing second language (L2) learners with the ability to communicate effectively in real-life social contexts. Sociopragmatics refers to the way conditions of language use derive from the social situation (Crystal, 2008).

Over recent decades, speech acts have been a major focus of research on students’ pragmatic competence (e.g., Alerwi and Alzahrani, 2020; Chang and Ren, 2020; Taguchi and Li, 2020); however, none of them considered the width of categorization of cognitive style, which makes our study original and fills a research gap.

## 3 Materials and methods

The research was carried out at Constantine the Philosopher University in Nitra at the Faculty of Arts and the Faculty of Education during the years 2014–2017.

### 3.1 Participants

The research was carried out at Constantine the Philosopher University in Nitra at the Faculty of Arts and the Faculty of Education during the years 2014–2017. The research sample consisted of 53 male and 95 female 21.5-year-old university students of the first, second, or third year of study. The students majored in English as part of a teaching program or translation studies program. They had studied English for 9 years and passed secondary grammar school examinations in English as a foreign language, tests ISED 3 level B2. Based on the C-W score, the students were divided into three groups: a narrow categorizer (score range from 0 to 55, *n* = 73), a medium categorizer (score range from 55 to 65, *n* = 45), and a broad categorizer (score range from 65 to 120, *n* = 30). In our study, the *max* C-W score was 98, *min* C-W = 7, *LQ* = 44, *UQ* = 64, *SD* = 1 4.37, *median* = 56, and the *mean* = 54.25.

Our participants (148 students) come from a high-context culture, are predominantly female, and tend to categorize cognitive style more narrowly rather than broadly.

### 3.2 Procedure

We have simulated two social situations for students of the English language. Both simulations of social situations included communication between a student and a professor, but one with social power and distance, and the second with social power and proximity (e.g., a request for an urgent call from a professor’s office, whom you know, and asking a professor, who is unknown to you for literature sources for research study). We used a discourse completion test to ensure cross-cultural comparability. The test (Diáz-Peréz, 2005) is composed of five socially differentiated situations, which vary in terms of the interlocutors’ relationships (dimensions of dominance or social power and social distance or familiarity). For our purpose, we have chosen two socially different situations in the context of the request.

Students were required to prepare appropriate responses to both social situations in the context of the request, i.e., students modeled the requests in the foreign cultural environment (English culture), in the contexts of social power distance and social power proximity.

We examine the occurrence of 30 social, language, and expressive factors in two situations (social distance D+ vs. proximity D- or with power P+), which were proposed by Díaz-Pérez (2003):

S2 (P+, D-): *You are in the office of one of your professors and you find out you desperately need to make a phone call. You cannot use any other phone than his/hers so you ask your professor to lend you his/her phone in his/her office*.

S3 (P+, D+): *You are preparing for your presentation for one of the most important subjects and you find out there is a new professor at the department who is an expert exactly in the field you are studying. You do not know that professor but you decide to see her/him and ask her/him to read the résumé of your presentation and to give you some advice for literature to study*.

We used transaction/sequence models for text representation, which allowed us to explore the relationships between the examined attributes and search for patterns/associations/rules among the identified elements in texts. Association rules were used for analyzing human behavior when formulating a request in a target culture affected by social distance/proximity.

In total, we obtained 148 different requests for each situation. Each request was manually analyzed by two linguists to ensure objectivity.

To examine the students’ cognitive style of category width (C-W), we used the C-W estimation scale by Pettigrew (1958).

### 3.3 Methods

#### 3.3.1 Request

We examined the occurrence of external and internal factors (social, language, expressive) in the requests modeling. Trosborg (1995) defines a request as a speech act whereby a requester conveys to a requestee that she/he wants the requestee to perform an act, which is for the benefit of the requester. Blum-Kulka et al. (1989) defined three elements of a request sequence in addition to the Head Act: alerters, supportive moves (external modifiers), and internal modifications. They stress the function of alerters to alert the requestee’s attention to the upcoming speech act.

We based our study on the typology of requests (Trosborg, 1995) and the theory of speech production (Blum-Kulka et al., 1989). We focus on the following 30 factors, which are divided into three groups—social, language, and expressive.

The first five factors represent alerters, the following four represent perspectives (in our study—social factors), factors F10–F19 represent internal modifications (syntactic and lexical/phrasal downgraders—language factors), and the rest cover external modifiers (supportive moves—expressive factors). This typology helped us create and specify language models of students in a foreign language.

##### 3.3.1.1 Social factors

(F1) Title or social role (e.g., Mr., Mrs., Doctor, Professor); (F2) Surname or friendly appellation (e.g., Mr. Smith, Mate); (F3) Name (e.g., Sarah); (F4) Attention getter (e.g., Excuse me, please); (F5) Combination of previous; (F6) Indirect perspective—allusion; (F7) Listener’s perspective (e.g., Could you); (F8) Speaker’s perspective (e.g., Could I); and (F9) Mixed perspective.

##### 3.3.1.2 Language factors

(F10) Negative formulation (e.g., I was wondering if you could not help me …); (F11) Present tense continuous; (F12) Modal verb question; (F13) Conditional; (F14) Imperative; (F15) Past tense; (F16) Other tenses or ways; (F17) Combination of previous elements; (F18) Correctness of an utterance (in terms of a grammatical structure); and (F19) Appropriateness of an utterance (in terms of culture specifics).

##### 3.3.1.3 Expressive factors

(F20) Politeness marker (e.g., Thank you, please); (F21) Pre-sequences/preparatory (elements before the core of a request, e.g., Hello Mary, I wasn’t at school yesterday, I felt sick so I stayed at home. Can you please lend me…); (F22) Post-sequences/supportive reasons (elements after the expressed request, e.g., Could I use your phone? It is very important for me and I have no other phone on hand.); (F23) Mitigating devices/disarmers (elements expressing an apology for disturbing, e.g., Sorry for interrupting, I remembered that …); (F24) Minimizers (elements minimizing the impact of a request, e.g., I would like to ask you for a small favour …); (F25) Consultative mechanism (e.g., Do you think I can take a shot of your notes?); (F26) Compliments/sweeteners (elements intensifying the likelihood of a request fulfillment, e.g., Could you help me prepare for my essay as I know you are an expert in the subject.); (F27) Intensificators (e.g., important, quick); (F28) Promises, reciprocity (Would it be o.k. if I borrowed the book for half an hour to photocopy a couple of chapters?); (F29) Combination of previous; and (F30) Other.

#### 3.3.2 C-W estimation scale

It examined the selected cognitive style, as well as real estimation. Jurčová and Sarmány-Schuller (1993) adapted the scale for the purpose of research in the Slovak context. The scale consisted of 20 affirmations referring to realia in the form of average value, and the respondent had to estimate which of the fixed numerical alternatives were related to the highest and to the lowest case of the selected phenomenon occurrence (one number is ticked in point A and the other in point B). The tasks were not predominantly testing knowledge but estimation of responses. The score was a sum of the numbers of responses with the highest and lowest estimations (A + B), and the higher the value, the broader the category width.

#### 3.3.3 Association rules

Association rule analysis is a technique that helps us discover the relationships between the examined items, i.e., find frequent patterns, associations, or correlations among examined items in the sets of translations. In our study, they help us find frequent patterns when a request is formulated, and how examined factors were associated with each other (similar to market basket analysis, a basket = a request).

There are three main measures of rule interest, which represent the strength of the rule:

1) *Support* (How frequently factor A is used. It is a proportion of transactions in which an individual factor A appears):

$$\mathrm{support}\;\left(A\right)=\frac{\mathrm{frequency}\;\mathrm{of}\;\left(A\right)}{\mathrm{number}\;\mathrm{of}\;\mathrm{transactions}\;\mathrm{in}\;\mathrm{the}\;\mathrm{dataset}}*100.$$

2) *Confidence* (How likely factor A is used when factor B is used in request modeling):

$$\mathrm{confidence}\left(\mathrm{if}\;A\;\mathrm{then}\;B\right)=\frac{\mathrm{support}\;\left(\mathrm{if}\;A\;\mathrm{then}\;B\right)}{\mathrm{support}\;\left(A\right)}*100.$$

3) *Lift* or *Interest* or *Correlation* (How likely factor A is used when factor B is used while checking for how frequently factor B is used):

$$\mathrm{lift}\;\left(\mathrm{if}\;A\;\mathrm{then}\;B\right)=\frac{\mathrm{confidence}\;\left(\mathrm{if}\;A\;\mathrm{then}\;B\right)}{\mathrm{support}\;\left(B\right)}\text{.}$$

We interpret the importance of a rule with the lift value. A lift value greater than 1 means that factor B is likely to be used if factor A is used, while if a value is less than 1 it means that factor B is unlikely to be used if factor A is used.

## 4 Results

We analyzed the occurrence of social, language, and expressive factors (F1-F30) in two different situations: S2 (P+, D-) and S3 (P+, D+), separately for narrow and broad categorizers.

**Figure 1:**
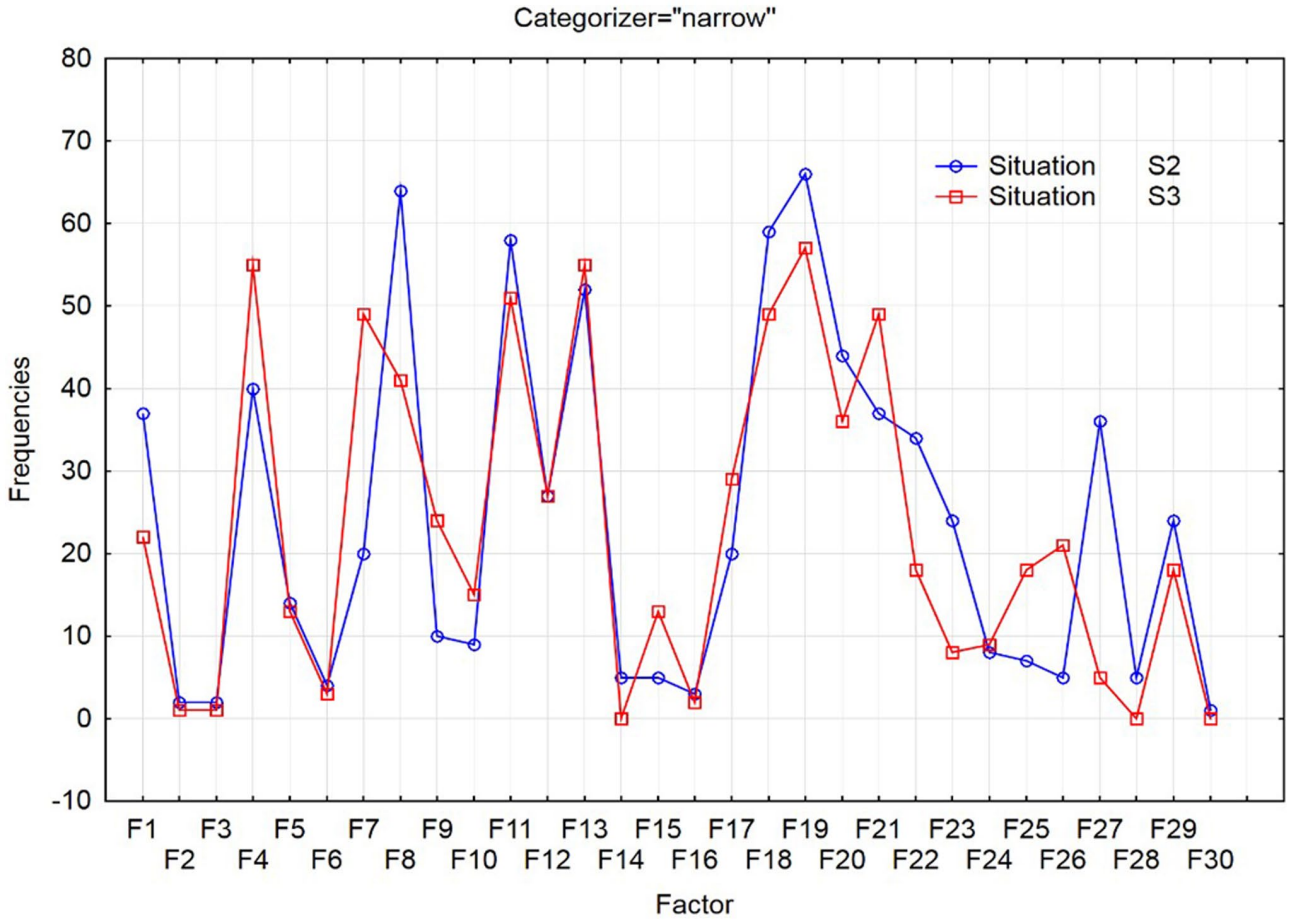
Occurrence of examined factors for narrow categorizers in situations S2 and S3.

**Figure 2:**
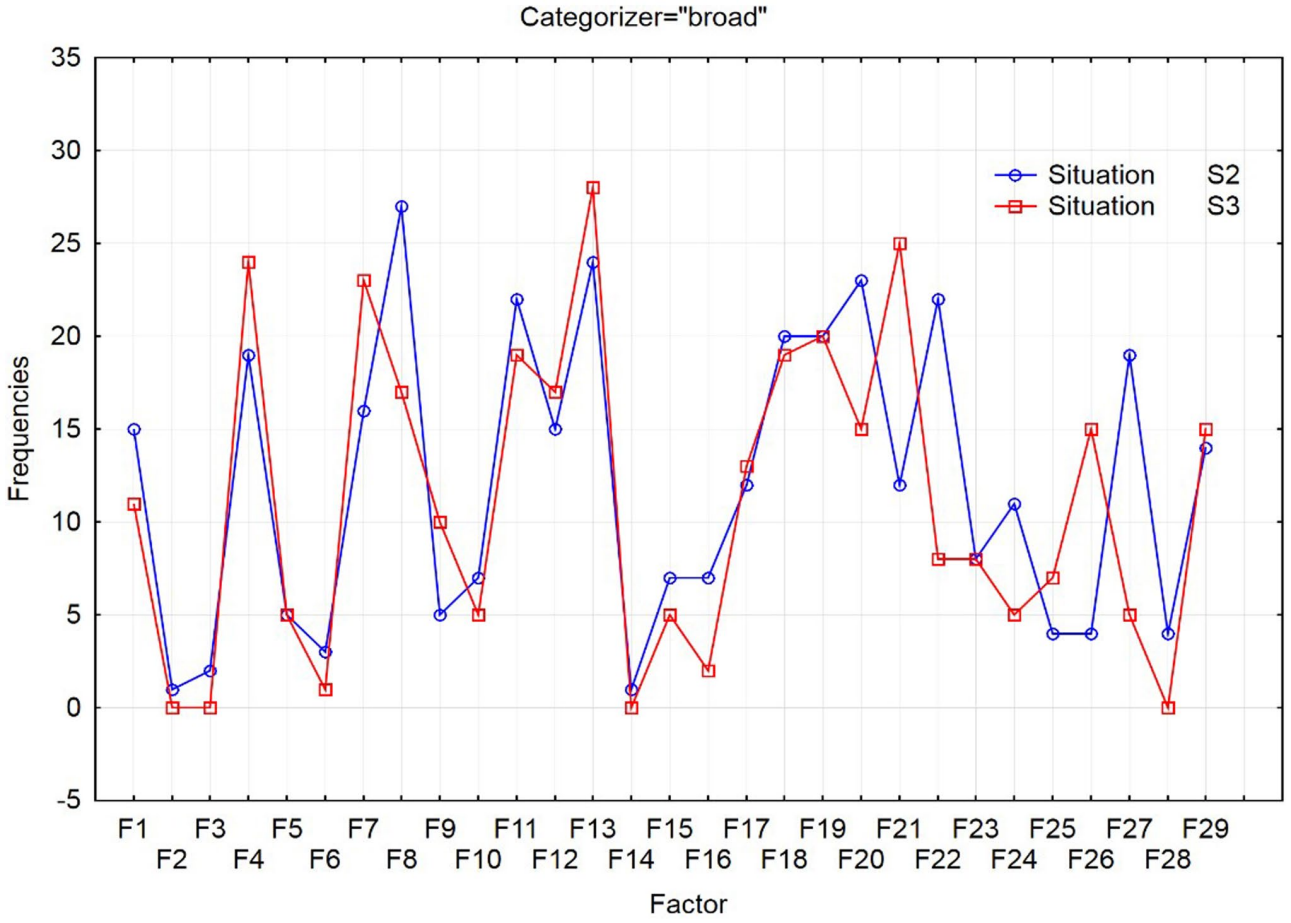
Occurrence of examined factors for broad categorizers in situations S2 and S3.

The differences between the created models of foreign language competence of the narrow categorizers depending on the power distance can be observed in Figure 1 and of the broad categorizers in Figure 2.

Based on the results of the chi-square test (*Pearson’s chi-square* = 103.7701, *df* = 29, *p* = 0.0000), the occurrence of examined factors of request modeling is related to social distance for narrow categorizers. We identified statistically significant but small correlations among the used factors in request formulation.

Similar to broad categorizers, based on the results of the chi-square test (*Pearson’s chi-square* = 48.9706, *df* = 28, *p* = 0.0084), the occurrence of examined factors of request modeling is affected by social distance.

Based on these findings, we decided further to distinguish the situations (S2 and S3) and category width (narrow and broad categorizers) when searching for associations among identified factors in request formulation.

### 4.1 Situation S2—social power and proximity (P+, D-) in relation to category width and foreign communicative competence

The narrow categorizers within her/his foreign communicative competence mainly use factors of appropriateness of an utterance in terms of culture specifics (F19) and also F8, F18, F11, F13, F20, and F4 (*support* > 54%). The factor F19 (appropriateness of an utterance in terms of culture specifics) has occurred in 66 requests, which represents 90% of examined requests, i.e., factor F19 was used 66 times in a request formulation (Table 1).

**Table 1:** Frequency of used factors in situations of social power and proximity (S2) for narrow categorizers.

Frequent factors	Frequency	Support (%)
(F19)	66.00	90.4109
(F8)	63.00	86.3013
(F18)	59.00	80.8219
(F11)	58.00	79.4520
(F13)	52.00	71.2328
(F20)	43.00	58.9041

The most popular pair of examined factors was appropriateness of an utterance in terms of culture specifics and speaker’s perspective (F19, F8; *support* = 79.45%) and then (F18, F19), (F8, F18), (F11, F19), (F8, F21), and (F8, F11) (*support* > 68%). Moreover, if the requester used politeness marker, she/he was likely to have used appropriateness of an utterance (in terms of culture specifics) in modeling the request (F20== > F19), as well; the factors F13== > F19, F18== > F8, F20== > F18, F19== > 18, F13== > F8, F8== > F20, and F19== > F8 occur in sets of factors more often together than as separate units (*lift* > 1). The confidence indicates how reliable the rule is (Table 2).

**Table 2:** Found association rules for narrow categorizers in situation S2.

Body	==>	Head	Support (%)	Confidence (%)	Lift
F20	==>	F19	57.5342	97.6744	1.0803
F19	==>	F13	67.1232	74.2424	1.0422
F18	==>	F8	72.6027	89.8305	1.0408
F18	==>	F20	49.3150	61.0169	1.0358
F19	==>	F18	75.3424	83.3333	1.0310
F13	==>	F8	63.0137	88.4615	1.0250
F8	==>	F20	52.0547	60.3174	1.0239
F19	==>	F8	79.4520	87.8787	1.0182

The broad categorizers (Table 3) use factors speaker’s perspective (F8) the most frequently, as well as F13, F11, F22, F18, F19, and F20 (*support* > 66%), i.e., factor F8 has occurred in 26 requests, which represents 86.66% of examined requests. The most popular pairs of examined factors were (F8, F13), (F8, F22), (F8, F19), (F8, F11), and (F13, F22) (*support* > 63%). In the case of rule using (*lift* > 1), factors F19== > F18, F27== > F8, F22== > F13, F4== > F13, and F27== > F13 occur in sets of factors more often together than as separate units (Table 4).

**Table 3:** Frequency of used factors in situations of social power and proximity S2 for broad categorizers.

Frequent item sets	Frequency	Support (%)
(F8)	26.00	86.6666
(F13)	24.00	80.0000
(F11)	22.00	73.3333
(F22)	21.00	70.0000
(F18)	20.00	66.6666
(F19)	20.00	66.6666

**Table 4:** Found association rules for broad categorizers in situation S2.

Body	==>	Head	Support (%)	Confidence (%)	Lift
F19	==>	F18	56.6666	85.0000	1.2750
F27	==>	F8	56.6666	100.0000	1.1538
F22	==>	F13	63.3333	90.4762	1.1309
F4	==>	F13	50.0000	88.2353	1.1029
F27	==>	F13	50.0000	88.2353	1.1029
F19	==>	F8	63.3333	95.0000	1.0961
F20	==>	F11	53.3333	80.0000	1.0909
F22	==>	F19	50.0000	71.4286	1.0714
F20	==>	F22	50.0000	75.0000	1.0714
F13	==>	F8	73.3333	91.6667	1.0576

**Figure 3:**
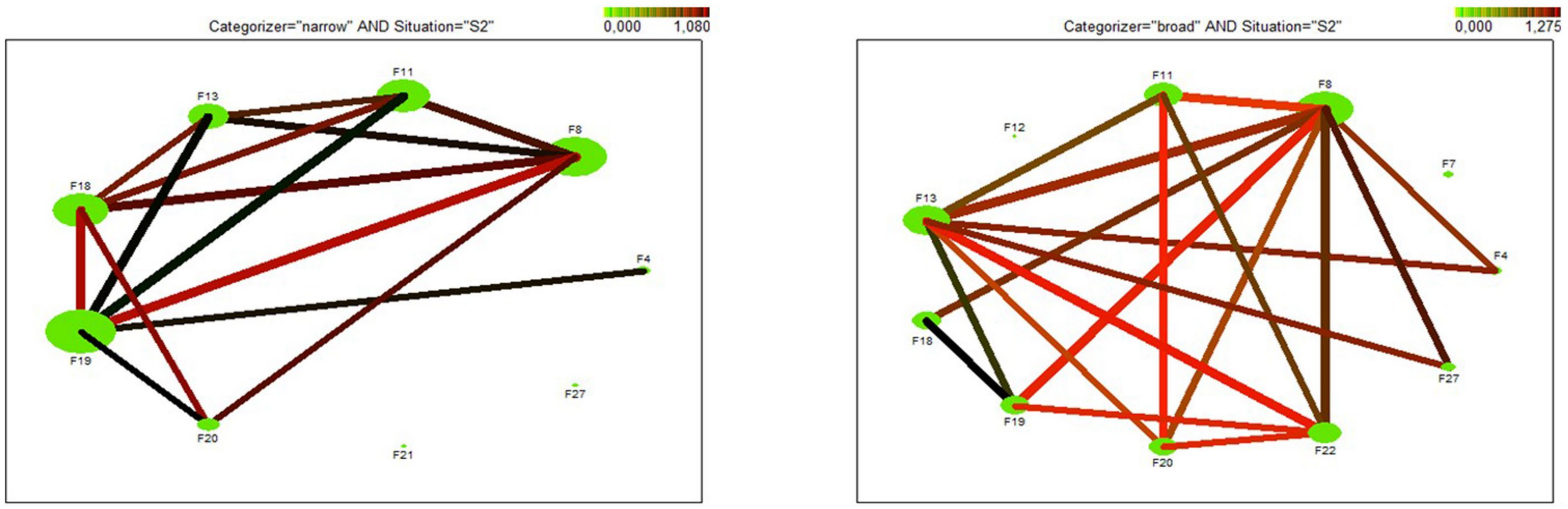
Web graph – visualization of discovered rules in situation of social proximity. a) narrow categorizers, b) broad categorizers.

The web graph (Figure 3) depicts discovered association rules for the request in the situation of social proximity. The node size represents the support of the occurrence of the factor, the thickness of the line represents the support of the pairs/combinations of factors, and the darkness of the line presents a lift of the rule.

### 4.2 Situation S3—social power and distance (P+, D+) in relation to category width and foreign communicative competence

The same association rules analysis was applied to the situation of social distance (D+), separately for narrow and broad categorizers.

Students of narrow category (Table 5) used the most factors, such as appropriateness of an utterance in terms of culture specifics (F19) and F4, F13, F11, F18, F7, and F21 (*support* > 68%). The most popular pair of examined factors was the appropriateness of an utterance in terms of culture specifics and attention getter (F19, F4) and then (F13, F19), (F4, F13), (F18, F19), and (F4, F21) (*support* > 67%).

**Table 5:** Frequency of used factors in situations of social distance (S3) for narrow categorizers.

Frequent item sets	Frequency	Support (%)
(F19)	57.00	89.0625
(F4)	55.00	85.9375
(F13)	55.00	85.9375
(F11)	50.00	78.1250
(F18)	49.00	76.5625
(F7)	48.00	75.0000

If the requester used the speaker’s perspective, she/he was likely to have used present tense continuous in the request, as well; the factors F8== > F11, F21== > F4, F13== > F21, F19== > 18, F13== > F21, F8== > F4, and F21== > F11 (lift >1) occur in sets of factors more often together than as separate units (Table 6).

**Table 6:** Found association rules for narrow categorizers in situations of social distance (S3).

Body	==>	Head	Support (%)	Confidence (%)	Lift
F8	==>	F11	57.8125	92.5000	1.1840
F4	==>	F21	67.1875	78.1818	1.1371
F19	==>	F18	75.0000	84.2105	1.0998
F21	==>	F13	64.0625	93.1818	1.0842
F8	==>	F4	57.8125	92.5000	1.0763
F19	==>	F21	65.6250	73.6842	1.0717
F19	==>	F4	81.2500	91.2280	1.0615
F7	==>	F21	54.6875	72.9166	1.0606
F13	==>	F4	78.1250	90.9090	1.0578

We found different association rules for models of problem-solving in speech production in a broad category.

The most popular factors (Table 7) were conditionals (F13) and then F4, F7, F21, F19, F11, and F18 (*support* > 70%). The most common associations were attention getter and conditional (F4, F13) and then (F7, F13), (F13, F21), (F4, F21), and (F4, F7) (*support* > 74%).

**Table 7:** Frequency of used factors in situations of social distance (S3) for broad categorizers.

Frequent item sets	Frequency	Support (%)
(F13)	26.00	96.2963
(F4)	24.00	88.8888
(F7)	23.00	85.1851
(F21)	22.00	81.4814
(F19)	20.00	74.0740
(F11)	19.00	70.3703

If a requester used a combination of expressive factors, she/he was likely to have used a combination of language factors in the request, as well (F29== > F17). Another factor (*lift* > 1) F18== > F19, F29== > F18, F29== > F19, F8== > F11, and F26== > F21 occurred more often together in transactions of used factors than separately (Table 8).

**Table 8:** Found association rules for broad categorizers in situations of social distance (S3).

Body	==>	Head	Support (%)	Confidence (%)	Lift
F29	==>	F17	48.1481	86.6667	1.8000
F19	==>	F18	70.3703	95.0000	1.3500
F18	==>	F29	51.8518	73.6842	1.3263
F19	==>	F29	51.8518	70.0000	1.2600
F11	==>	F8	55.5555	78.9474	1.2538
F21	==>	F26	51.8518	63.6364	1.1454
F26	==>	F4	55.5555	100.0000	1.1250
F7	==>	F20	48.1481	56.5217	1.0900
F8	==>	F21	55.5555	88.2353	1.0828
F7	==>	F13	85.1851	100.0000	1.0384

**Figure 4:**
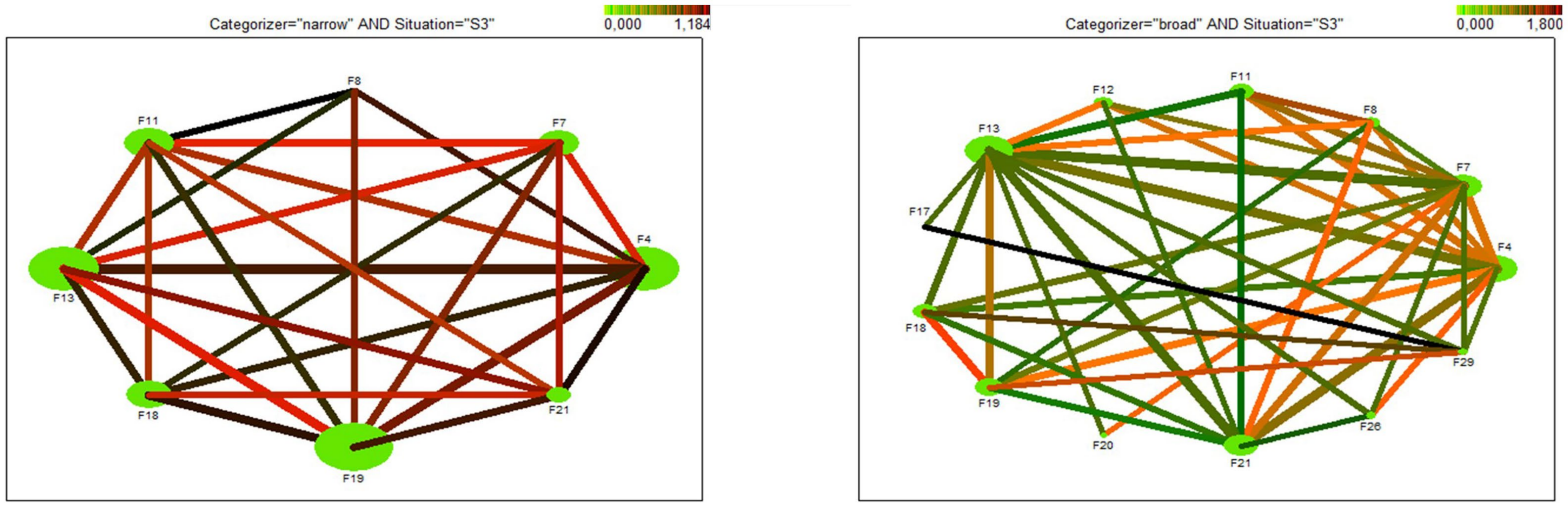
Web graph – visualization of discovered rules in situation of social distance. a) narrow categorizers, b) broad categorizers.

The web graph (Figure 4) depicts discovered association rules for the request in situations of social distance.

## 5 Discussion

We will interpret our results in two lines. The first is focused on the impact of social power and distance on foreign communicative competence, which is related to the first two research questions from the introduction.

It was statistically proved that formulating a request in a foreign language—foreign language competence—found by narrow and broad categorizers, is related to social distance. Broad and narrow categorizers create certain models when formulating a request, which are affected by social distance, i.e., they apply rules using certain relations, link selected social, language, and expressive factors in request formulating in a situation of social distance or proximity (D- or D+). Students, regardless of the width of the categorization, in a situation of social distance prefer to address the stranger by title rather than first name or last name or friendly appeals and to use an attention getter, such as Excuse me …, in a greater extent and address the request in “I” perspective, such as Could I … (speaker’s perspective). These findings correspond with Hofstede and Hofstede (2017), and high-context cultures, such as Slovak, accept inequality of power positions in society and show respect to authorities. Equality is preferred in English-speaking countries as depicted also in the English language, but in a situation of social distance, foreign communication competence of an EFL student is influenced by her/his mother tongue and culture, which was also demonstrated by Ezzaoua (2020).

Within language factors, correctness and appropriateness of an utterance have the greatest influence on foreign communicative competence in terms of meaning, greater than the use of conditionals in requests. This finding is in line with Glaser and Limberg (2020), and lexical and grammatical knowledge does not influence communicative competence as regards the appropriateness of language use in context. According to Adnyani and Suwastini (2022), students use more complex sentences rather than simple questions, which also correspond with the social power that is distinguished by forms of address, e.g., someone with power, such as professor and doctor, and expects to be addressed more formally.

Among the expressive factors, it is more diverse. In a situation of social distance, pre-sequences have the greatest influence, i.e., preparatory elements before the core of request, and vice versa, in the situation of social proximity, students prefer post-sequence elements, i.e., elements expressed after a request. Intensificators and compliments, and/or sweeteners have a similar effect, which confirms the finding of Alsallal et al. (2020) that compliments are used more often in situations of social proximity than of social distance. Similarly to Napoli and Tantucci study (2022) focusing on request patterns formulated in English and Italian, factors such as mitigators and intensifiers are influenced by social distance.

To conclude, foreign communicative competence is affected by social distance, mainly in the use of social and expressive factors, such as titles (social role) and attention getter, but mainly the listener’s and speaker’s perspective, pre- and post-sequences, and intensificators. This corresponds to Biesenbach-Lucas (2007) claims that messages addressed upward (status of addressor and addressee) are frequently more formal and polite, and more conforming with conventional norms, which has an impact on the choice of social, language, and expressive factors. Our findings relate to Díaz-Pérez (2003), who also proved the relationship between the use of factors of speech production and social distance and power.

The second line of interpretation of our results is related to the width of categorization of cognitive style and its impact on foreign language competence in the context of social distance, which is related to two other research questions.

Social distance has been shown to have an impact on foreign language competence, both in narrow and broad categorizers. In terms of alerters, which are used to initiate a conversation or attract the requestee’s attention, we showed their occurrences to be equal for both narrow and broad categorizers. The dominant alerters in both widths of categorization included the attention getters, such as please or excuse me, and social role/title, such as professor. However, in the case of social distance, both widths of categorization used attention getters to start or initiate a request more often than in the case of proximity; on the other hand, both categorizers used alerter, such as the title, more often in the case of social proximity than of distance. This corresponds with Maros and Halim (2018); students adopt the norms of the given social group and environment when greeting and initiating a conversation. It is also supported by Savic (2014) claiming that alerters used in initiating a conversation follow the rules and norms, which are based on social distance. Maros and Halim (2018) are of the opinion that EFL students are likely to transmit their native social and cultural norms into the target language. Regarding the perspective of formulating the request (Could I/Could you), EFL students of both categories tend to formulate from the “I” perspective when it comes to a request to a professor they already know, but in the case of a stranger (an unfamiliar) professor they prefer to formulate a request from the “you” perspective. This finding partially corresponds with Biesenbach-Lucas (2007), and the non-native students formed the majority of their requests in emails in English from the expected perspective (“you”), except for the appointment requests; in the case of appointment requests, students chose to express their requests from their own perspective (“I”). We suppose that both situations, writing emails and requesting professors, are related. If one asks someone for a meeting, there is a high probability that one knows her/him and formulates the request from the “I” perspective.

One finding of interest is the influence of the width of categorization of cognitive style on linguistic factors, specifically, internal modifications of the head act as syntactic and lexical/phrasal downgraders, and/or elements that may reduce the degree of imposition of request. According to Warga (2005), in the majority of requests, students (native and non-native) prefer the form of the conditional to the present tense and other linguistic factors that represent the request head act. In our research, this is confirmed only for broad categorizers, regardless of social distance, contrary to narrow categorizers, mainly in a situation of social proximity.

Another remarkable finding within syntactic and lexical/phrasal downgraders is that while the broad categorizer uses linguistic factors in terms of grammatical structure and in terms of cultural specifics to the same extent, regardless of social distance, the narrow categorizer differentiates between social distance and proximity. She/he prefers appropriateness to correctness, using them more frequently in situations of social proximity than of distance. The broad categorizer in the situation of social proximity shows the tendency to be polite rather than concentrate on a formal aspect, which is amplified by intensifying the urgency of the situation (intensifier), by supporting reasons and explaining the cause of the request; however, she/he concentrates on him/herself and the appropriateness of the request to fulfill her/his requirements. There is a strong link between the appropriateness and correctness of a request, but we suppose that the broad categorizer perceives the grammatical correctness of the used language factors globally rather than in detail (the use of the form of conditioning does not associate it with any grammatical tense, as in the case of a narrow categorizer) and does not analyze the grammar structure more deeply (she/he uses conditional but does not associate it with another linguistic factor compared to narrow categorizer). A narrow categorizer is characterized by an effort to be cautious, rigid, and secure in cognitive decision-making (Sarmány-Schuller, 2011), and for this reason, she/he focuses primarily on the appropriateness and correctness of his/her utterance and increases the request using the conditional.

A similar interesting finding was shown with external modifications of the head act, such as supportive or mitigating moves (expressive factors)—in the case of using minimizers (elements minimizing the impact of a request) and mitigating devices. The broad categorizer used mitigating devices equally, regardless of social distance, hoping that the professor would comply with her/his request. However, the narrow categorizer used mitigating devices to varying degrees, more frequently in the situation where she/he knows the professor (proximity) than in the situation of social distance. The extent of minimizers use is also a remarkable paradox. It is precisely opposite to mitigating devices. A narrow categorizer used minimizers to the same extent, regardless of social distance, while a broad categorizer used minimizers to varying degrees but more frequently in a situation of social proximity. While the narrow categorizer used minimizers to the same extent, the broad categorizer used minimizers depending on social distance, and vice versa, while the broad categorizer used mitigating devices to the same extent, the narrow one used mitigating devices depending on social distance.

Through association rules, we can describe the lexical and syntactic choice of both narrow and broad categorizers depending on social distance.

In the case of social distance, if the narrow categorizer chooses an attention getter (F4), such as Excuse me …, then with 78% probability she/he uses preparatory elements (F21) before the request itself, and with 93% probability she/he chooses the conditional form (F13), while with 72% probability she/he will formulate the request to the requestee from the listener’s perspective (F7). If he/she places emphasis on appropriateness (F19), then there is 84% probability that correctness is emphasized, as well (F18).

The second most likely possibility of how a narrow categorizer formulates her/his request in a situation of social distance is that if she/he formulates the request in the “I” perspective (F8), such as Can I …, then with 92% probability she/he uses the present tense (F11) or attention getter (F4). If she/he chose attention getter, then the request will copy the model mentioned above.

If the broad categorizer decides to form the request from the requestee’s perspective (F7), he/she will use the conditional with 100% probability, but only with 56% probability of the politeness marker (F20), such as please. However, if she/he used the present tense (F11), she/he would use the speaker’s perspective (F8) with 78% probability and then preparatory elements (F21) with 88% probability. However, if she/he used compliments intensifying the likelihood of a request fulfillment (F26), she/he would use an attention getter (F4) with 100% probability. If she/he places emphasis on appropriateness (F19), then with up to 95% probability correctness is also emphasized (F18).

In the case of social proximity, if the narrow categorizer chooses the politeness marker (F20), then with 97% probability appears the intention of appropriateness (F19) but only with a 61% probability of grammatical correctness (F18). Moreover, this occurs with only a 60% probability of formulating the request from the speaker’s perspective (F8). If she/he uses the “I” perspective, she/he uses the conditional (F13) with 88% probability.

If a broad categorizer uses the conditional (F13), then with 90% probability she/he would use supportive reasons, while she/he uses the speaker’s perspective (F8), such as Could I …, and with 88% probability she/he will use an attention getter (F4) and intensifiers (F27). If she/he uses the “I” perspective form, then she/he will emphasize appropriateness with 95% probability. This finding is supported by Kamińska (2014) who claims that in foreign language learning, broad categorizers frequently over-generalize the rule or create generalizations.

## 6 Conclusion

Although we have clearly defined communicative competence, whether in the mother tongue or in a foreign language, there are no definitions or even extensive studies that would define how to be communicatively competent in a given situation since communicative competence differs not only at a cultural level but also at a social and personal (individual) level, which was also confirmed in our research.

The contribution to the field of research consists of answering our four questions set out in the introduction. *To what extent is communicative competence affected by power distance (D- or D+) in a foreign culture, and/or to what extent does student’s communicative competence reflect power distance (D- or D+) in a foreign language?* Our results revealed that power distance influences the foreign communicative competence of a student. Requests addressed in an environment of social power and distance (a student asking a professor, whether she/he can make a phone call from his office or a student asking a professor to provide her/him with resources for writing her/his essay) are affected by the student–professor relationship. A requestee (student) initiates a conversation following the rules and the norms of her/his mother tongue and culture, which are based on social distance. Since the Slovak culture belongs to low-context culture, foreign communicative competence is even more influenced by the hierarchical relationship of the requester and requestee. The requester tends to be more formal and polite and transfers conventional norms of the culture of the mother tongue (source culture) into English, which mainly affects the use of alerters and external modifications of the head act of request.

As for answering the last two questions regarding the impact of category width on foreign communicative competence, specifically *to what extent the foreign communication competence is affected by the width of categorization (narrow vs. broad) in target low-context culture* and *to what extent the foreign communication competence reflects the student’s width of categorization (narrow vs. broad) in the English language—*our research indicates a certain degree of dependence among internal modifications of the head act of request (syntactic and lexical choice) and category width and also external modifications of the head act request (elements that may reduce the degree of imposition of request) and category width.

A broad categorizer, regardless of social distance, prefers to formulate the request in a conditional over the present tense form, contrary to narrow categorizers, who in a situation of social proximity prefer the request form in the present tense. A similar finding was shown in the case of external modifications of the head act, where we observed the inversion between broad and narrow categorizers, mainly in the use of minimizers (elements minimizing the impact of a request) and mitigating devices.

The limits of our study comprise the unequal representation of narrow and broad categorizers. Out of 148 students, we included only 73 broad and 30 narrow categorizers in our research. Students who achieved an average C-W score or values close to the average were excluded from the analysis. The second factor that could have influenced our results is the method of data collection. Students recorded their statements in written form, i.e., they wrote their requests, as they would say them. The third factor remains in the manual labeling of requests. Manual labeling of 30 individual factors was carried out by one evaluator. Her/his subjectivity could affect the results of the research. In future, we plan to repeat the experiment with a larger sample of participants and use modern IT tools for data collection and text pre-processing to create a corpus of requests in a foreign language and subsequently, through corpus-based methods, to confirm or not our results.

An interesting secondary finding in our research is the fact that teaching training or translation and interpreting training are more likely to be studied by women who tend to narrowly categorize cognitive styles. They prefer detailed analytical information processing, which can be related to the study of a foreign language, which can be the subject of further research in the field of psychology of language.

## Data availability statement

Datasets used and/or analysed during the current study are available from the corresponding author on reasonable request.

## Ethics statement

Ethical review and approval was not required for the study on human participants in accordance with the local legislation and institutional requirements. Written informed consent from the patients/participants OR patients/participants legal guardian/next of kin was not required to participate in this study in accordance with the national legislation and the institutional requirements.

## Author contributions

DM: Writing – original draft. ES: Writing – original draft. MM: Writing – original draft.
