# Supplementary material for: Communication models in a foreign language in relation to cognitive style category width and power distance
Source: Front Psychol. 2024 Jan 8;14:1272370. doi: 10.3389/fpsyg.2023.1272370 (PMC10800717; doi:10.3389/fpsyg.2023.1272370)
Supplement: Supplementary file 1 [file Data_Sheet_1.docx]

Supplementary Material

Communication models in a foreign language in relation to cognitive style category width and power distance

Dasa Munkova*, Eva Stranovska, Michal Munk

*** Correspondence:** Dasa Munkova: dmunkova@ukf.sk

# Supplementary Data

# Supplementary Figures and Tables

## Supplementary Figures


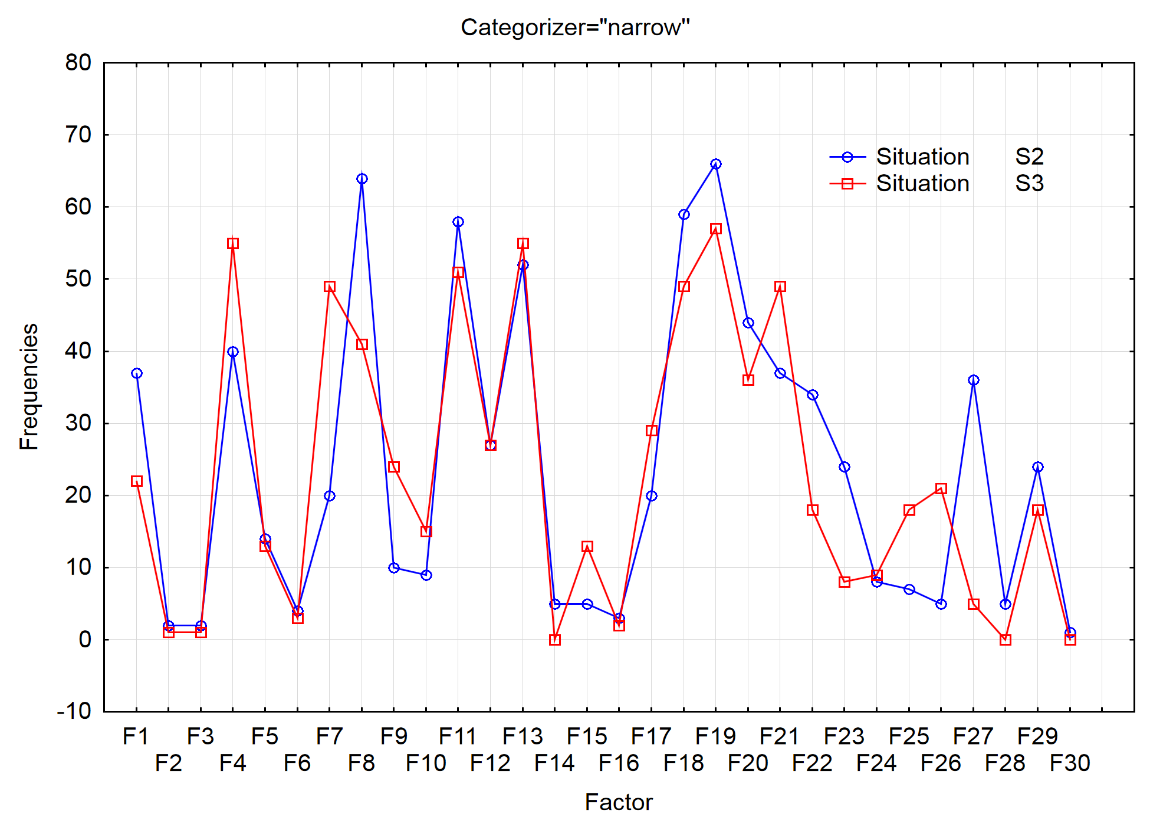


**Supplementary Figure 1.** Occurrence of examined factors for narrow categorizers in situations S2 and S3.


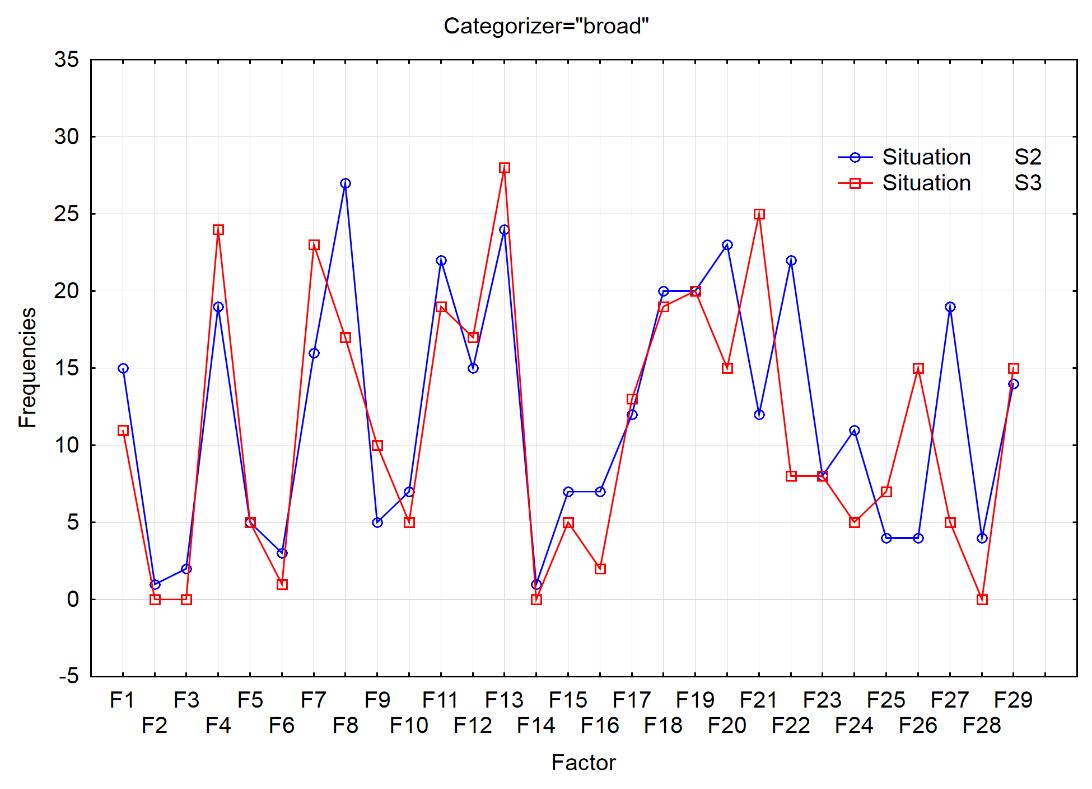


**Supplementary Figure 2.** Occurrence of examined factors for broad categorizers in situations S2 and S3.


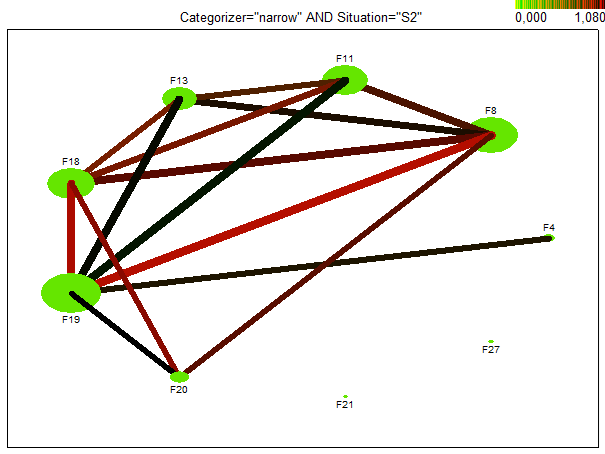

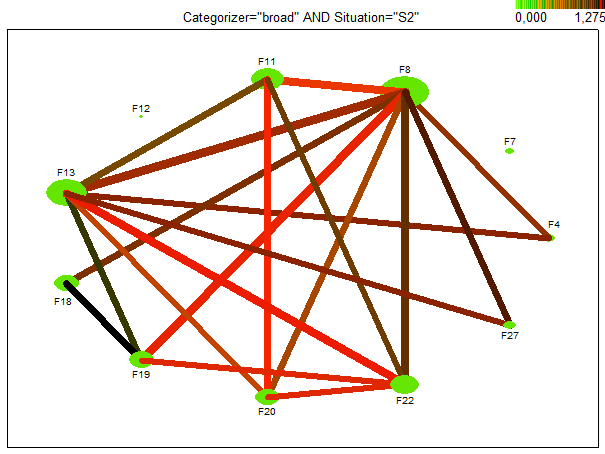

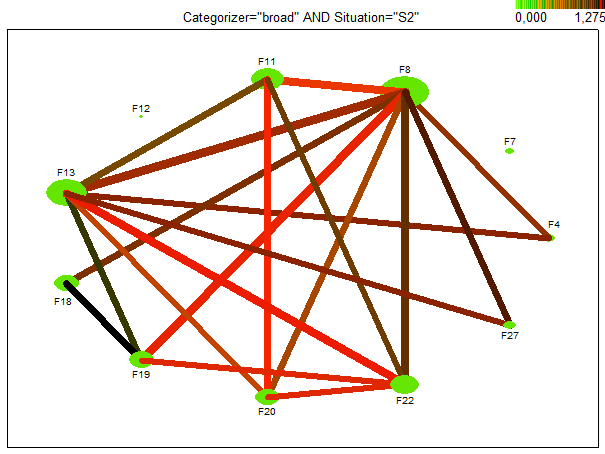

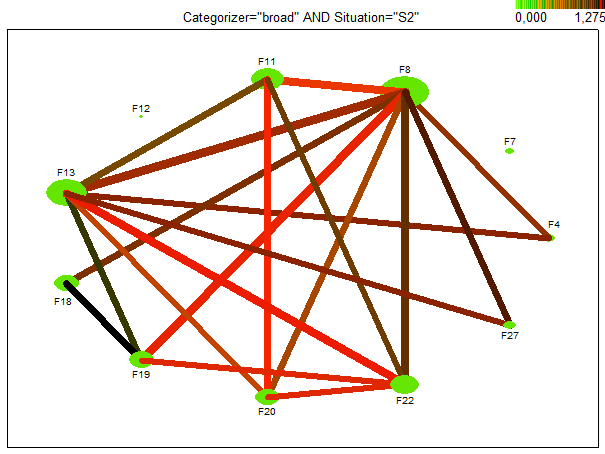


**Supplementary Figure 3.** Web graph – visualization of discovered rules in situation of social proximity a) narrow categorizers, b) broad categorizers.


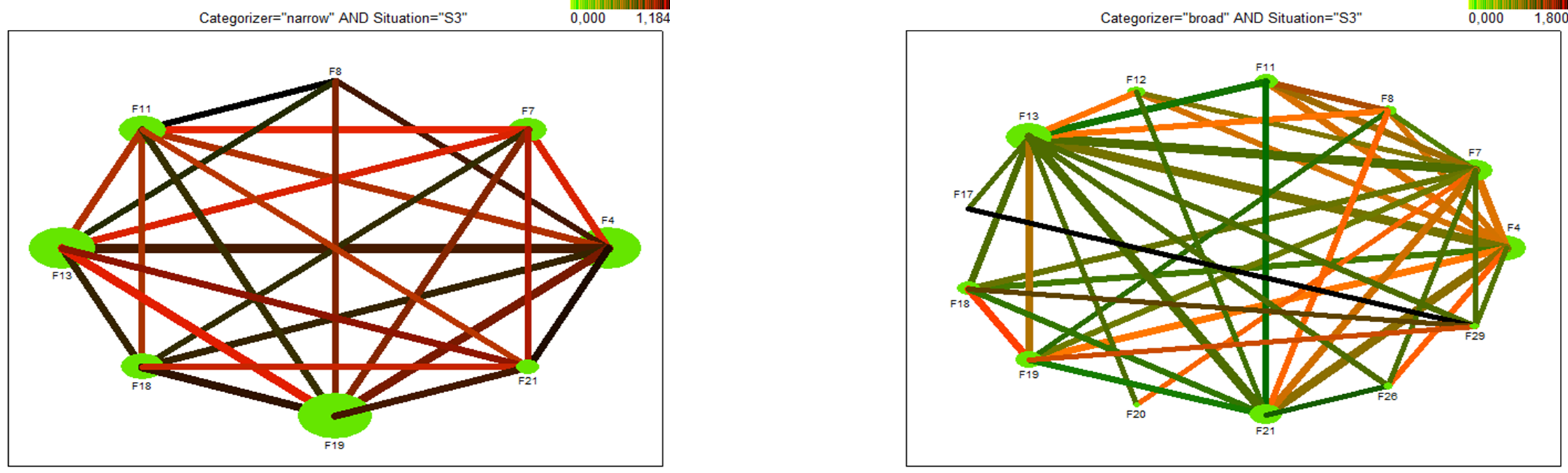


**Supplementary Figure 4.** Web graph – visualization of discovered rules in situation of social distance a) narrow categorizers, b) broad categorizers.
